# How to be a very safe maternity unit: An ethnographic study

**DOI:** 10.1016/j.socscimed.2019.01.035

**Published:** 2019-02

**Authors:** Elisa G. Liberati, Carolyn Tarrant, Janet Willars, Tim Draycott, Cathy Winter, Sarah Chew, Mary Dixon-Woods

**Affiliations:** a THIS Institute (The Healthcare Improvement Studies Institute), University of Cambridge, UK; b Department of Health Sciences, University of Leicester, Leicester, UK; c Women and Children's Health, North Bristol NHS Trust, Bristol, UK

**Keywords:** UK, Qualitative, Patient safety, Maternity care, Ethnography, Evaluation

## Abstract

Maternity care continues to be associated with avoidable harm that can result in serious disability and profound anguish for women, their children, and their families, and in high costs for healthcare systems. As in other areas of healthcare, improvement efforts have typically focused either on implementing and evaluating specific interventions, or on identifying the contextual features that may be generative of safety (e.g. structures, processes, behaviour, practices, and values), but the dialogue between these two approaches has remained limited. In this article, we report a positive deviance case study of a high-performing UK maternity unit to examine how it achieved and sustained excellent safety outcomes. Based on 143 h of ethnographic observations in the maternity unit, 12 semi-structured interviews, and two focus groups with staff, we identified six mechanisms that appeared to be important for safety: collective competence; insistence on technical proficiency; monitoring, coordination, and distributed cognition; clearly articulated and constantly reinforced standards of practice, behaviour, and ethics; monitoring multiple sources of intelligence about the unit's state of safety; and a highly intentional approach to safety and improvement. These mechanisms were nurtured and sustained through both a specific intervention (known as the PROMPT programme) and, importantly, the unit's contextual features: intervention and context shaped each other in both direct and indirect ways. The mechanisms were also influenced by the unit's structural conditions, such as staffing levels and physical environment. This study enhances understanding of what makes a maternity unit safe, paving the way for better design of improvement approaches. It also advances the debate on quality and safety improvement by offering a theoretically and empirically grounded analysis of the interplay between interventions and context of implementation.

## Highlights

• Maternity care continues to be a site of avoidable serious harm.
• An ethnographic case study identified mechanisms linked to maternity safety.
• Context and intervention are co-constitutive: neither alone is fully explanatory.
• Structural influences on safety are of enduring relevance.

## 1 Introduction

Improving newborn and maternal health is increasingly high on the global health agenda (WHO, 2017), but the safety of maternity care remains problematic worldwide. Avoidable harm in maternity services results in serious disability and profound anguish for women, their children, and their families (Andreasen et al., 2012), and imposes substantial burdens on health systems, including the cost associated with litigation (Magro, 2017). Though much has been learned from inquiries and investigations into adverse events in maternity care (Kirkup, 2015; Knight et al., 2017; Kurinczuk et al., 2014), the resulting recommendations have not, as in other areas of quality and safety (Card et al., 2012), always had impacts that are as powerful or consistent as might be hoped. Evidence-based approaches to making improvement are thus much needed (Dixon-Woods and Martin, 2016).

Two major approaches to improvement may heuristically be distinguished, based largely on their underlying logics. One draws on a logic of intervention. Here, a specific, bounded intervention (such as a checklist, decision-support system, or care bundle) is defined and implemented. This approach lends itself to evaluative study designs aimed at determining the impact of the intervention on pre-specified outcomes (Portela et al., 2015). Such studies may produce evidence of the average effects of an intervention, but are less good at explaining why variability in those effects occur in different places. When the role of context is discussed, it is usually of interest for how it might modify the effects of an intervention, often described using a “barriers and facilitators” discourse (Szymczak, 2018).

The second approach is based on a logic of context. Identifying the features of particular environments (such as organisational structures, processes, behaviours, practices, and values) that contribute to safety, it is the approach used, for example, in studies of high reliability organisations, as reported by Weick and Sutcliffe (2001):

*(1) Preoccupation with failures rather than successes, (2) reluctance to simplify interpretations, (3) sensitivity to operations, (4) commitment to resilience and (5) deference to expertise, as exhibited by encouragement of a fluid decision-making system. Together these five processes produce a collective state of mindfulness.*

The focus on context as generative of safety underpins the recent turn from a Safety-I paradigm (preoccupied with fixing adverse events that have already occurred) to Safety-II (focusing on system flexibility and resilience, as well as behaviours and processes that produce successful practice) (Hollnagel, 2014). However, a major challenge for approaches that urge the reproduction of features of context is that they lend themselves much less easily to traditional evaluative techniques, inhibiting the development of the kind of evidence-base that may be most persuasive to clinical and policy audiences. A further risk is that, if applied at too high a level of abstraction, research about particular contexts may simply reproduce high-level thematic categories that are already well-known – such as leadership, culture and use of data – without being sufficiently informative and specific about what precisely needs to be done in order to achieve the same results elsewhere (Vincent and Amalberti, 2016).

A challenge for both approaches is that the dialogue between them has remained limited, in part because of epistemological divides that fail to unite accounts of specific interventions aimed at improvement and accounts of contexts that might be generative of improvement (Dixon-Woods, 2014; Howarth et al., 2016). Yet, as some have begun to argue, particularly in the public health field (Rutter et al., 2017) much is likely to be gained by a complex systems approach that recognises the ways that interventions and contexts are co-constitutive and mutually emergent.

In this article, we examine the role of both the contextual features and a specific intervention in contributing to excellence, using a positive deviance (Lawton et al., 2014) case study of a high-performing maternity unit. Positive deviance studies seek to produce learning by examining individuals, teams, or organisations that show exceptionally good performance. However, the forces that create positive conditions for safety may be at least partially invisible to those who create them because they remain tacit or habitualised. In-depth, qualitative examinations of positively deviant settings are thus needed (Lawton et al., 2014).

We focus our analysis on a maternity unit at Southmead Hospital in Bristol, UK. Using an operational definition of safety as the control and management of risk over time to maximise benefit and to minimise harm (Vincent and Amalberti, 2016), Southmead can be seen as an example of positive deviance when measured by its rates of birth complications, which are among the lowest in the UK published literature (Draycott, 2013). Additionally, the unit has reported improvements on a range of clinically relevant perinatal outcome measures over a sustained period from 2001, including a 100% reduction in permanent brachial plexus injuries (Crofts et al., 2016), a 50% reduction in both hypoxic ischaemic encephalopathy and low 5-min Apgar score (Draycott et al., 2006), a 50% reduction in the time taken to expedite birth in potentially life-threatening cases of umbilical cord prolapse (Siassakos et al., 2009), and improved composite neonatal outcomes, including a reduction in the rate of neonatal intensive care admission from 38% to 22% (Siassakos et al., 2009). Consistent with a more positive view of safety (Hollnagel, 2014), high performance is also evident in staff attitude surveys indicating a positive safety culture, outstanding teamwork climate, and high job satisfaction (Siassakos et al., 2011).

Using a logic of intervention, these improvements might be attributed to a training programme known as PROMPT (Practical Obstetric Multi-Professional Training) (Box 1), which was developed at Southmead in 2000, and has been continuously implemented, refined and updated since. However, one reason for caution in attributing improvement solely to PROMPT is that reliably propagating the success seen at Southmead has not been straightforward. For example, the implementation of PROMPT in an Australian state was associated with some improvements, but the results were modest compared with those seen in Southmead, and not all sites were able to implement the programme as intended (Shoushtarian et al., 2014). This suggests a need to understand the contextual features of Southmead that might be generative of safety, while retaining the possibility that PROMPT may nonetheless have an important role.

Box 1 **What is the PROMPT programme?**

PROMPT (PRactical Obstetric Multi-Professional Training) is an evidence-based programme developed to reduce adverse neonatal and perinatal outcomes through local multi-professional training. A systematic approach to improving maternity safety, PROMPT encompasses knowledge and skills training, emergency simulation, and systems improvement.

The training is typically run *in situ* and focuses on both the technical and non-technical skills required to manage different types of obstetric emergency, emphasising the importance of effective communication, teamwork, and interdisciplinary work. Training is envisaged to be compulsory for all maternity staff, led by local trainers, and bringing multi-professional clinical teams together in their normal working environment to rehearse, reflect and improve on their collective practice (Macrae and Draycott, 2016).

The current approach for scaling and spread is the train a trainer model (T3), which entails small groups of doctors and midwives from different maternity units attending a one-day training event led by the main PROMPT faculty. The T3 days, conducted in a non-clinical setting, include demonstrations of how to run PROMPT locally and provide guidance and support materials (the *PROMPT Course in a box*) to support the implementation of training in local maternity units.

Alt-text: Box 1

In this article, we aim to characterise what makes Southmead safe, attending both to features of context and intervention to generate an in-depth understanding.

## 2 Methods

### 2.1 Context

Our analysis of context is limited to the maternity unit clinical micro-system (Mohr et al., 2004); the hospital and wider policy and structural context are outside the scope of this study. Southmead Hospital (Bristol, UK) has a large maternity unit (approximately 6500 births per year) serving some of the most deprived areas of Bristol. The midwife-to-birth ratio is currently 1:30. Our access was facilitated by two local collaborators (in senior positions in the unit, and co-authors on this paper – TD and CW), who helped in ensuring staff and parents were informed about the study and were happy to engage.

Attending the PROMPT programme annually is compulsory for all staff working in the unit, including midwives (maternity unit, community, birth centre, and locum midwives), consultant obstetricians, obstetric anaesthetists, maternity and healthcare assistants (MCAs and HCAs), maternity theatre staff, and doctors in obstetric training. Systems are in place to release staff from clinical activities to secure 95% attendance of all staff groups.

#### 2.1.1 Data collection

We adopted an ethnographic approach (Hammersley and Atkinson, 2007), involving observations, semi-structured interviews, and focus groups. To offer an in-depth, longitudinal and recursive analysis not tied to specific individuals who happened to be on duty or specific events, data for the study were collected in two phases: between September 2014 and March 2015 and between July and August 2017. This approach allowed analytic depth by enabling exploration and probing of issues identified in early observations and interviews.

Three researchers (JW, EL, CT) conducted approximately 143 h of observations of between one and three days (including a night shift) in the maternity unit. They also observed two PROMPT in-house training events at Southmead and one national PROMPT train-the-trainers event. Observations were open-ended (a predetermined observation checklist was not used), but, consistent with our research aims, particular effort went into capturing how routines, interactions, social norms and structures contributed to or interfered with safety. For observations, oral consent was obtained from participants, who were informed about the researchers’ background as social scientists.

Two researchers (JW and EL) conducted 12 semi-structured interviews with nine clinicians working in the unit (five doctors and four midwives, some of whom were involved in the local PROMPT programme), and with three individuals with a management or risk management role.

Both in our field conversations and in interviews, we adopted a maximum variability strategy to access as many different points of views as possible on the issues of interest. Participants were selected in consultation with the local collaborators to represent a range of different disciplines and seniority levels, and were approached by the researchers during the fieldwork. Our recruitment process was guided by the principle of information power (Malterud et al 2016), which shifts the focus from the number of participants included in a sample to the power of information the sample holds.

Interviews focused on participants’ understanding of the mechanisms that contributed to, or hindered, safety in the unit, as well as allowing follow-up on aspects of practice seen in observations. Interviews lasted 60–120 min and were conducted face-to-face or by telephone. JW also conducted two focus groups, one involving midwives and MCAs, and one with consultant obstetricians, anaesthetists, and doctors-in-training. Interview and focus group participants signed a consent form. The study received ethical approval from the London – Harrow Research Ethics Committee.

#### 2.1.2 Data analysis

Ethnographic data were captured both in the form of *in situ* fieldnotes and audio-recorded debrief reflections that were recorded by individual researchers at the end of each day's observations. Research team debriefs were conducted to enable reflexivity and synthesis, and were audio-recorded. All audio-recorded material (interviews, individual debriefs, and group debriefs) were transcribed verbatim, anonymised, and included in the analysis. Documents collected on site visits were also treated as data.

A systematic and iterative approach to analysis of the observation and interview data was based on the constant comparative method (Charmaz, 2014). A selection of transcripts was open-coded by EL and SC. Key themes were identified through repeated close readings and review of open codes. This allowed us to produce a coding framework, which was used to conduct (and was updated throughout) the analysis of the remaining transcripts. Analysis was enhanced by drawing on a range of theoretical resources and sensitising concepts that we identified as relevant and useful in moving from description to interpretation, including concepts from the sociology of work (e.g. normalisation of deviance, jurisdictions and professional boundaries), organisational theory (e.g. situated learning theory, legitimate peripheral participation, collective sense-making), and safety science (e.g. resilience and high reliability, distributed cognition). As data collection and analysis proceeded concurrently, we were able to discuss our preliminary findings with participants and to use their feedback to test our emerging analysis against their perceptions. QSR NVivo software was used to aid the coding, management and retrieval of data.

## 3 Results

We identified a range of mechanisms that contributed to safety in Southmead's maternity unit (Table 1). Our findings demonstrate how safety emerges through an interplay between context and the intervention (the PROMPT programme).

**Table 1:** Mechanisms implicated in the high performance of the maternity unit at Southmead Hospital.

Mechanism	Observable indicators
Collective competence	• Interdependency, collegial behaviours, and strong social ties among staff • Mutual respect across roles and disciplines • Disagreements settled through open discussion rather than personal or positional power • Care organised around the shared goal of safe childbirth, with professional boundaries managed flexibly • Sapiential authority (Boreham, 2004): deference to expertise rather than hierarchy
Insistence on technical proficiency	• Expectation of very high standards of proficiency in clinical tasks • High-fidelity training to develop technical competence • Informal training and role modelling in routine care delivery (e.g. clinical cases discussed during handovers or informal conversations) • Learning through legitimate peripheral participation (Lave and Wenger, 1991)
Monitoring, coordination, and distributed cognition	• Mechanisms and roles allocated to maintaining a shared awareness of the external situation in the maternity unit • Staff in coordinating roles playing a control room function (Roe and Schulman, 2018) • Constant effort to ensure that the team is fit to cope with the circumstances
Clearly articulated and constantly reinforced standards of practice, behaviour, and ethics	• Values and standards are clear, articulated, and reinforced through role modelling • E.g., safeguarding the dignity, safety and psychological wellbeing of women and family is paramount • Social control: individuals take actions to ensure that other people behave in a way that is aligned with the unit's standards
Monitoring multiple sources of intelligence about the unit's state of safety	• Data are used to sense problems • Hard indicators: routine clinical data are constantly scrutinised, updated, and made available to all staff • Soft intelligence: use of patient complaints and staff ground knowledge to learn and improve safety • Psychological safety: staff can raise safety concerns without fear of embarrassment, retaliation, or punishment
Highly intentional approach to safety and improvement	• Commitment towards safety is collectively pursued and socially legitimised (not externally imposed) • Organisational citizenship behaviours: discretionary effort to promote the safety and effective functioning of the unit • Combination of formal risk management (i.e. allocated roles and formal activities, such as safety checks) and embedded risk management (frontline clinicians proactively preparing for risky situations and detecting small signs of deterioration)
Structural influences on mechanisms for safety	• Staffing levels • Financial resources • Physical infrastructure • Equipment • Clinical complexity

### 3.1 Collective competence

A defining feature of the unit was its *collective competence:* the kind of collective performance that cannot be disaggregated into the sum of individual competencies, but instead conforms to three normative principles: making collective sense of events in the workplace, developing a sense of interdependency, and developing and using a collective knowledge base (Boreham, 2004).

Several elements contributed to collective competence. The PROMPT training days supported the development of social relationships and a sense of interdependency, providing an opportunity for staff to learn together and to develop their understanding of each other's roles and responsibilities, as well as confidence in each other's technical skills. Through the simulations, staff from different disciplines and roles developed a shared mental model of the procedures for dealing with emergencies: they could clarify mutual expectations, experiment with different leadership and followership styles, pick up on each other's social cues, and appreciate the valuable roles of all staff, including porters and Maternity Care Assistants (MCAs).

*The consultant said that the outcome for maternity patients really is dependent upon the team understanding the whole [process of care delivery]. She said that part of it is due to how they'd all trained together, they'd all been on the same course, they'd all been indoctrinated with the same belief. (Observation, labour ward, 2017)*

The values and aspirations of the unit were not formulated as ceremonial mission statements, nor were they imposed from the top. Instead, various members of staff (midwives, doctors, MCAs, risk managers, administrators, and others) consistently expressed a mutual sense of “us”: they described a powerful feeling of belongingness, identified with the unit's values, and experienced high levels of pride.

Shared goals across disciplines and professions were an especially strong feature of collective competence on the unit. For instance, the shared goal of “safe birth” helped to avert interdisciplinary tensions.

*That's what makes Southmead different: there is true multi-professionalism. That culture of teamwork now … is embedded in the way we work on a daily basis without even realising that we do it. (Consultant obstetrician, 2017)*

Effort went into socialising junior members into an ethos of collective endeavour and mutual respect across roles and disciplines. For example, medical students were assigned to a midwife mentor (rather than a doctor) during their professional training. Most doctors were seen to listen carefully and respectfully to midwives, with disagreements settled through open discussion rather than deployment of personal or positional power.

*The midwife coordinator and a senior obstetrician were trying to make a plan for a woman, and there was a little bit of a discussion about her, because the obstetrician really wanted to bring [the woman] up to the central delivery suite right away. But [the obstetrician] then actually listened to the midwife coordinator and agreed to go down to the day assessment unit to look at the lady beforehand. (Observation in the labour ward, 2017)*

This and other observations suggested that hierarchies were managed flexibly and strategically: they were mobilised when there was need for clarifying social norms and behavioural standards, but they were muted when not required. Consistently, actions taken did not necessarily depend on professional hierarchies but rather on the requirements of the situation. Some senior doctors were, for example, willing to take on assistant or comforting roles when their clinical input was not required.

*We were doing all the correct things [to manage a post-partum haemorrhage] … And then a consultant … strolled in the room, he must have had the view we were getting on alright, but he picked up that the father was, as you can imagine, shaking in the corner, and so he just came in and comforted the father. (Midwife coordinator, 2015)*

Faced with an emergency, there was a collective acceptance that, rather than organisational or professional hierarchies taking precedence, the person with the most relevant expertise would lead the situation. Although the different responsibilities of each profession were acknowledged, the principle of sapiential authority – authority based on experience, or having particular information or skills (Boreham, 2004) – was observed. Supporting collective competence were also strong norms of cooperation and collegiality, reciprocal ties, and personal investment.

*Everybody knows everybody, you can be yourself, there's no kind of airs and graces. […]. It's a nice atmosphere in which to work. (Obstetrician, 2015)*

*One of the midwives had just started her break. She looked really shattered. Another midwife popped her head into the room and said “Can anyone help me with a Synto[cinon] infusion?” And the first midwife, who had literally just stopped, said “Well, my tea will need to cool down anyway”, and she just jumped up and followed the second midwife in one of the rooms. (Observation, staff room, 2017)*

Crucial to supporting healthy relationships was the multidisciplinary staff room. Used flexibly by doctors, midwives, and other staff, it was a break-out space that blurred boundaries between working and socialising, and meant that working relationships were injected with friendship, mutual trust, and an authentic desire to help. The result was that people knew each other well: they were aware of their colleagues’ skills, strengths and weaknesses, wellbeing, and ability to cope. They made decisions and took action accordingly.

*We [have] one communal coffee room for doctors and midwives and so those kinds of conversations that you have with people – the silly conversations … The personal things that people share, give you insight into that person and perhaps how they work, and I think having the ability to understand people … does help. (Midwife, 2015)*

### 3.2 Insistence on technical proficiency

The unit was characterised by an unrelenting insistence on technical competence: individuals were required to be able to perform their clinical tasks to a very high standard of proficiency. The effects were visible in staff confidence, readiness, and competence when undertaking complex procedures and responding to crises (Macrae and Draycott, 2016).

*Somebody had a cardiac arrest whilst in labour … That [was] the most stressful thing that I have ever done in my whole life … But I didn't doubt, I didn't think …, I just did it. So, like the first five minutes I was in complete drill mode and it's just like “This is what we have to do and this is what we're doing”. (Locum consultant obstetrician, 2015)*

The mandatory PROMPT programme was one important way in which technical proficiency was achieved. Using interactive teaching techniques, simulations with high quality mannequins, patient actors, real-life equipment, and home-made props, the sessions were conducted with as much fidelity as possible to real-life clinical practice. The focus was on learning through doing, enabling direct experience of the feel of techniques and the practical skills of assembling and using equipment. Drills and simulations were typically followed by feedback from the participants and peer-observers to encourage critical reflection and collective sense-making.

*During the neonatal resuscitation session, the PROMPT trainers were getting people to try and use the manual respirator on the baby mannequin. Everyone took turns and it was really about feeling how you place the mask over the baby's mouth, about where you rest the respirator on your arm, about how fast you squeeze it, how you do five breaths with a big gap. (Observation, PROMPT training day, 2014)*

These formal training events were far from the only opportunity for fostering technical proficiency. During our observations, senior staff were highly visible on the shop floor and worked to achieve a balance between supporting the learning of juniors and allowing them to make autonomous decisions. For example, in setting clear standards for what were considered appropriate actions and decisions, they explained not just how but why, allowing colleagues to develop their skills through reflection-in-action (Argyris and Schön, 1978). Junior staff were supported to develop their skills by participating in lower-risk tasks; we observed numerous examples of this legitimate peripheral participation (Lave and Wenger, 1991), which ensured that junior staff not only increased their proficiency but also became acquainted with the skills, vocabulary, and principles of the community.

*The midwife coordinator asked the newly qualified midwife, “Have you done any suturing yet?” She said no. The coordinator then said to her, “Well if you get a small tear, the best thing you can do is to suture it yourself but get me to come and watch you do it”. (Observation, staff room, 2015)*

Conversations were also important. Knowledge-sharing at the backstage (Waring and Bishop, 2010), based on situational opportunity, was common: clinical cases were frequently discussed during handovers, ward rounds, or in informal conversations.

### 3.3 Monitoring, coordination, and distributed cognition

Assuring the safety of the unit and securing its preparedness required the constant monitoring of the functional state of the system. Deliberate effort went into anticipating and mitigating stresses, and facilitating shared awareness and systems of distributed cognition (Lingard, 2009). Critical to this was a *control room* facility, which Roe and Schulman (2018) characterise as a coordinating structure that seeks to manage a critical service reliably and safely in real time.

In Southmead, this structure was achieved through explicitly charging staff in specific roles (such as the midwife coordinator) with monitoring both the unit's internal situation (e.g. its ability and readiness to cope with the rapidly changing circumstances) and the external situation (e.g. patient flow, match of staffing to need, communication between clinical settings, and potential risks emerging from capacity pressures). This was highly skilled work done under enormous pressure; it required being aware of clinical situations that could deteriorate at short notice and reallocating staff accordingly using knowledge of expertise and clinical circumstances.

*The midwife coordinators here are fantastic … They really have to know the skill-mix inside out, know exactly who they need to support and when, and they're thinking about the bigger picture, you know, they're not just coordinating [the] labour ward, they have to be aware of the issues on the postnatal wards, know when there are beds coming up, who needs to come up from the antenatal wards, who's coming in from outside … I think part of the reason this place works so well is testament to them. (Registrar, 2017)*

Frontline clinicians – both doctors and midwives – were often observed planning ahead for anticipated problems or risky situations, aiding collective detection and awareness of hazards and ensuring that risks were mitigated.

*One of the midwives was expecting her lady to have a post-partum haemorrhage. She grabbed the post-partum haemorrhage trolley and put it right outside the room. She said “There's nothing I hate more than having to do everything at the last minute during an emergency, when you have to be in the room and you have to look after the woman and you don't have time to go and find what you need”. (Observation, labour ward, 2017)*

Staff were also aware of the importance of real-time monitoring of their immediate situation and unfolding events. Individuals were actively encouraged by their seniors to speak out in the moment if they identified an unseen risk in a situation, or had concerns about the potential for escalation of a problem, and were reassured that it was better to flag up any concerns than to keep quiet.

The work of monitoring, coordination, and response was aided by the unit's whiteboard, which was constantly updated to provide accurate information on labouring women as well as general safety information. Located in the multidisciplinary staff room, members of staff congregated around it frequently, both formally during the twice-daily structured handovers and informally throughout work shifts and breaks. Another important mechanism in monitoring of current state and coordination of response lay in the handovers, ward rounds, and board rounds that were compulsory for staff to attend and were conducted with diligence.

*We also have a really strong culture of doing regular ward rounds … so we're not fire-fighting, we're trying to anticipate what's going to happen and where risk is. (Registrar, 2017).*

### 3.4 Clearly articulated and constantly reinforced standards of practice, behaviour, and ethics

The unit was characterised by clear articulation and enforcement of standards of good practice, behaviour, and ethics. For example, though making mistakes was tolerated, hiding or ignoring them was not. Similarly, handovers, briefings, and safety checks were taken very seriously; staff knew they needed to arrive on time and that interruptions were unwelcome. PROMPT had a key role in clarifying and reinforcing these shared standards, including expectations of adherence to the unit's policies and use of local tools designed to protect safety. The training also played a role in socialising newcomers.

*The system stays the same and individuals come and go. And the individuals have to slot into a system. But they've got to be [ac]culturated into that system, and the training day does that. (Research midwife, 2015)*

We observed numerous instances of standards being articulated and enforced in day-to-day clinical practice. Through mentorship, role modelling and situated learning, staff members attempted to socialise newcomers into “how things are done here”. Senior staff constantly mobilised an image of the unit as high-performing, disciplined, and hardworking, which in turn generated expectations that staff would live up to shared standards.

The training days further served the important function of creating and curating collective memories, with adverse incidents being shared and reflected upon (Campbell and MacPhail, 2002; Guareschi and Jovchelovitch, 2004). In some cases, the sharing of emotions also appeared to be critical in helping to reinforce values of high standards of clinical practice and avoidance of error.

*In this case, the baby had [a serious condition] that should have been spotted, and everybody you could see was visibly upset. (Observation, handover, 2017)*

Core ethical standards were consistently reinforced, including that of ensuring a positive childbirth experience that safeguarded dignity. Staff strove to achieve these standards in their own practice, indicating value congruence (Cazier et al., 2007).

*The woman was close to delivery and she was in a lot of pain. The anaesthetist, the consultant obstetrician, the registrar, the SHO [senior house officer] and the midwife coordinator were in the room. The consultant wanted to assess her and the midwife coordinator asked everyone to step out. She said “There's nothing worse when you are in such pain then being examined in front of a gallery of people”. (Observation, labour ward, 2017)*

As observed in Bosk's classic ethnography, Forgive and Remember (2003), as well as other studies of healthcare settings (e.g. Tarrant et al., 2017), enforcement of standards relating to conduct and technical proficiency mainly took the form of social control. Individuals informally monitored each other's conformity to professional and social norms and used graduated sanctions to bring behaviour back in line. Minor problems were dealt with through talking to offending colleagues, often using humour or other social sanctions.

*There was one junior doctor who came in late for the doctors' handover. She was given some of the jobs to do that day that [nobody wanted]. Somebody laughed and said “Oh, enjoy your day” … It was light-hearted but it was like a punishment as well, it definitely made a point. (Observation, labour ward, 2015)*

Participants in the ethnography and interviews explained that non-adherence to established clinical standards was possible, so that clinical discretion was preserved, but had to be discussed and justified, not simply treated as an automatic privilege of status or position.

*Sometimes if locums come, we might not give them a good time actually, because if they think “I'm the consultant here I'm doing this”, and we're thinking, “Oh no you're not” … And it's not that we're being horrible, but … we would then say, “Well our guidelines say this, it's evidence-based. And this is our protocol and this is what we do”. (Midwife, 2017)*

Deviations from the unit's standards that were not perceived to be in good faith, or were seen as unintentional but persistent defaults, were actively managed through techniques such as escalation, keeping a close eye, or buddying. Staff were also prepared to intervene more forcefully when they felt the situation warranted it.

*[The locum consultant] was managing the delivery, I was the registrar in the room … and [he] was essentially pulling on this baby's head in front of me. I told him not to pull, and he kept pulling, so I sort of told him to stop and gently kind of pushed him out the way, and then took over and delivered the baby. That was quite a brave thing to do, but I knew that I was right … There's no point having that argument afterwards, because you could have a baby with a paralysed arm. (Registrar, 2015)*

### 3.5 Monitoring multiple sources of intelligence about the unit's state of safety

The unit was committed to monitoring multiple sources of intelligence about safety to identify where improvements could be made. The unit had configured, and was constantly innovating with, systems for gathering both hard data (e.g. metrics and measures) and soft intelligence (e.g. staff and patient feedback) (Martin et al., 2015, 2018; Turner and Pidgeon, 1997). An automated maternity dashboard was used to monitor performance by identifying any concerning trends or patterns. The data were discussed routinely in multiple forums and at the PROMPT training days, promoting collective responsibility for improvement.

*If there are things that come up on the dashboard then people, particularly the junior doctors, are encouraged to audit it to find out what the reasons are behind that and then sometimes it can just be their own initiative. (Registrar, 2017)*

Importantly, data were used to sense problems rather than to seek comfort (Dixon-Woods et al., 2014), indicating the kind of *chronic unease* described by high reliability scholars (Weick and Sutcliffe, 2001).

*I think we're good but we can always improve … We're probably good compared to some other units, but there's always room for improvement. (Focus group, 2015)*

Staff themselves were respected as an important source of intelligence about safety concerns: their “on the ground” knowledge was seen as an invaluable resource. The PROMPT training days were an important forum for using staff expertise to identify hazards and weaknesses in systems and processes. More generally, staff were encouraged to notice problems and operational failures (Tucker, 2004) and bring them to collective attention rather than tolerating or working around them (Hewitt and Chreim, 2015). Noticing took place both in real time and during more reflective periods.

*On the [white]board in the staff room, staff also write general safety things like, say for instance “We've only got one ventouse on the ward at the moment, let's find some others”, “The wheelchair that we have hasn't got the feet on, we must make sure that nobody uses that anymore because it's a safety hazard”. (Observation, maternity unit, 2015)*

Women on the unit were also often asked about their experiences, and complaints were routinely investigated and used in training. The unit also used creative ways to gain intelligence on current practice, including, for example, requesting clerical staff to observe ward rounds and using the feedback to inform improvements.

*The secretaries thought the ward round was not well organised … The consultant said that the secretaries had held a mirror up to them and … it was an opportunity to make it better. (Observation in the maternity unit, 2015)*

Critical to the unit's ability to gather soft intelligence, and to sense and resolve safety problems, was its active cultivation of a sense of psychological safety: staff could raise safety concerns and be confident that they would not face embarrassment, retaliation, or punishment (Edmondson, 1999). Senior staff described how they deliberately framed risk as inherent to clinical practice but controllable, and went to great lengths to ensure that all staff could seek help, both emotional and technical, when needed. We observed senior staff members openly discussing their own errors, making it normal to be honest about the precarious nature of clinical practice and the fallibility of individuals.

*[In the midwives' handover] the coordinator went on to talk about a lady that, when she came in, they really thought wasn't progressing at all, and they were planning to asking her to go back home. But the coordinator then said that, on a vaginal examination, it turned out she was actually six centimetres dilated. There were a few giggles and laughs at this and it was very light-hearted, but it was an opportunity to emphasise that you mustn't go by what you first think, and for the students to hear that a very senior midwife possibly got it wrong, that that can happen to everyone. (Observation in the maternity unit, 2015)*

### 3.6 Highly intentional approach to safety and improvement

Individually and collectively, staff demonstrated a highly intentional, deliberate approach towards safety. Rather than taking safety for granted, it was seen as something requiring constant vigilance and purposeful organisation. Accordingly, the unit was characterised by attention to *operational fitness* – ensuring that the workflow and systems were optimised for the tasks that teams needed to accomplish. Consistent with human factors principles, effort went into the design of equipment, space, and information technology with the aim of reducing cognitive load, enhancing convenience, and facilitating safety. For example, algorithms, toolboxes, and stickers were widely used to support consistent practice, and to “make the right way the easy way” (Consultant obstetrician, 2015).

Individual accountability and responsibility towards safety relied on staff identification with the unit's values and pride at being part of the Southmead team. Staff demonstrated organisational citizenship behaviours – behaviours that are discretionary in nature (not part of an individual's contractual tasks) and that promote effective organisational functioning (Organ et al., 2005). The PROMPT days clearly contributed to this by celebrating the unit's achievements and individual contributions to the collective good. Staff were consistently and explicitly encouraged and supported to take responsibility for making changes in day-to-day practice by fixing seemingly small problems (e.g. tools, objects, or procedures becoming obsolete or getting in the way of safety) before they created substantial risk. If a solution seemed effective, it was spread and scaled-up and was publicly praised. For example, the emergency boxes used on the unit, which contained all of the equipment needed to handle urgent clinical scenarios, had their origins in an idea from someone low in the formal hierarchy:

*It was actually one of the maternity care assistants that said to somebody “When we get an emergency, I'm running in and out of this room like a tennis ball, you know, why not put [all the tools] together so that I can just pick it all up in one thing?”. (Senior midwife, 2015)*

Formal risk management systems also played an important role in supporting safety on the unit, both in proactively identifying risks and putting plans in place to mitigate them, and in responding to hazards identified through intelligence-gathering. Great emphasis was placed on reflexive practice (Argyris and Schön, 1978) and having formal structures to support improvement.

*We have the safety brief … We had a post-partum haemorrhage forum, we've got clinical risk forums. We've got meetings … that just gives you the opportunity to take stock and sit down and discuss your professional opinion with professional people that you work with. (Senior midwife, 2017)*

A small team of risk-managers worked collaboratively with clinicians to design risk management strategies. Accordingly, for the most part, the administrative controls and workflows in the unit were accepted as sensible and facilitative of work rather than as irritating mandates imposed from above.

### 3.7 Structural influences on mechanisms for safety

Not everything at Southmead worked perfectly all the time. Sometimes things did not go smoothly: we witnessed a number of near-misses, some of them potentially serious; procedures were sometimes not followed; relationships occasionally frayed; and operational systems were sometimes imperfect or malfunctioned. Although the local PROMPT programme was maintained throughout our data collection, we noticed some deterioration in the mechanisms underpinning safety between our first set of observations in 2014–15 and our second set in 2017. This seemed to be associated with worsening structural conditions, including financial constraints and severe staff shortages, increasingly complex clinical situations, and deteriorating physical infrastructure.

*It feels increasingly like a battle to keep [the unit] safe. It feels like at the moment our heads are above water but it wouldn't take much for it not to be. (Registrar, 2017).*

The unit's resilience and ability to cope with adverse circumstances became particularly vivid in these conditions. We observed many striking examples of staff preserving safety via the mechanisms described above, including, for example, the midwife coordinator aiding collective situational awareness.

*During the handover, the midwife coordinator said to the rest of the team “I do need to warn you that today the corridor [linking the central delivery suite with the birth centre and NICU] is going to be closed from 10am to 2pm, because of plumbing works”. She said “I am going to make absolutely sure that the birth centre doesn't take any women that have even the slightest risk of having to be transferred to the central delivery suite”. (Observation, maternity unit, 2017)*

Adverse structural conditions (staffing, physical infrastructure, and so on) sometimes caused severe strains. They affected proactive risk management by hindering the team's alertness to small signs of deterioration, and caused exhaustion and stress as shortages meant staff were unable to take breaks during 13-h shifts. The increasingly challenging environment impacted on the whole system, creating frustration, sparking tensions between the trust management and frontline clinicians, affecting collegial and supportive behaviours, creating psychological distress, and reducing organisational citizenship behaviours. Maintaining safety was becoming increasingly dependent on the goodwill and commitment of staff, but was becoming stretched to its limits.

*[If we are still safe it is because staff] go above and beyond … Because we give so much and stay, and we would pull together. We have hit rock bottom in the last 18 months … Staff have been broken, upset, staff have been in tears, [it's been] massively challenging. (Senior midwife, 2017)*

## 4 Discussion

Using a positive deviance approach (Lawton et al., 2014), this study enhances understanding of the mechanisms that are important to achieving safety in maternity care. The study also advances the theoretical debate on quality and safety improvement by reflecting on the interplay between interventions and context of implementation, and empirically demonstrating their mutually constitutive nature.

### 4.1 Mechanisms contributing to safety at Southmead

Using the maternity unit at Southmead Hospital as a case study, our analysis has identified six interactive mechanisms that appear to be implicated in its outstanding safety performance (Table 1). A particular feature of the unit was the collective character of its standards of good clinical practice. Clinical work in the labour wards was highly interdependent, characterised by extreme uncertainties, surges, and resource constraints. Under such conditions, individuals' technical proficiency, perceived confidence in each other's skills, and proactive, formal, and informal risk management systems were key to securing safety. Equally important was the social organisation of work (Strauss et al., 1985). Strong social ties and mutual investment created a supportive and psychologically safe environment, but also generated rich tacit and relational knowledge (including individuals knowing each other's strengths and weaknesses), which, in turn, buttressed patient safety. Though divisions of labour persisted, they were highly functional; Southmead evaded the threats to safety linked to dysfunctional relationships between doctors and midwives that have surfaced in recent reports on high profile scandals in UK maternity units (Kirkup, 2015). Professional boundaries were managed in ways that supported effective response to the unit's contextual demands (Liberati, 2017), including unpredictability of the workload and complexity and acuity of the case mix. The unit was able to maintain a generative tension between nurturing a just culture (acknowledging the fallibility of individuals and the impact of faulty systems) and promoting individuals' agency and accountability for safety (a sense that individual behaviours can and will make a difference) (Aveling et al., 2016). Individual contribution was reinforced through a number of mechanisms, including the visible commitment of senior staff, recognition and reward of individuals' contribution, encouraging “noticing” behaviours, and responding to concerns raised.

These findings confirm that commitment to professional values should not be easily dismissed as purely self-serving (Freidson, 2001). They emphasise the need to go beyond encouraging effective communication simply as a set of techniques, to appreciating the relevance of the deeper and less visible properties of social relationships, and they further add to scholarship that conceives of competence as highly context-dependent and as the product of a social system, rather than residing in an individual body or mind (Hutchins and Klausen, 1998; Lingard, 2009).

A danger of the kind of analysis we present here is that safety is seen as solely cultural, behavioural, or organisational in character. It is important not to dismiss the relevance of structure as well as the wider political context. Consistent with models that conceive quality outcomes as dependent on *process* as well as *structure* (Donabedian, 1988), the six mechanisms we have identified are deeply influenced by structural conditions (e.g. staffing levels, the physical environment, and the wider organisational, social and political environment). These findings indicate that any intervention to improve safety may founder in inadequate structural conditions.

### 4.2 Bridging the gap between the ‘intervention’ and ‘context’ improvement logics

Failure to replicate and scale successful improvement interventions in healthcare is often attributed to either a lack of fidelity in implementing the original intervention, or to the nature and characteristics of the context in which the intervention is introduced. Though these approaches help identify some explanations for failure to improve, they do not, in contrast with recent developments in the public health literature (Rutter et al., 2017), grant sufficient attention to the multiple, complex, and dynamic ways in which interventions and contexts interact. Our study of how safety is achieved in a high-performing maternity unit provides a unique description of what these interactions look like in practice, and offers empirical support for the argument that contexts may “shape or co-construct complex interventions and therefore cannot be considered separately from those interventions” (Wells et al., 2012).

The six mechanisms we have identified were fostered and constantly reinforced through a specific safety intervention (i.e. the PROMPT programme). PROMPT facilitated the formation of strong social relationships and an ethos of openness and transparency; provided important opportunities for improving technical skills, communication, and teamwork competence; and meant that staff felt confident in noticing, and acting on, breaches of standards. But, in turn, the adoption and sustained implementation of PROMPT itself depended on the broader features of the unit, including the constant reinforcement of standards of good practice and a highly intentional approach towards safety and improvement. The unit functioned as a moralising entity, constantly articulating shared goals and collective endeavour, and using socialisation of newcomers and social control mechanisms as its primary means of enforcement to ensure the stability of standards and norms despite staff turnover. PROMPT was, on its own, far from a magic bullet: the programme interacted constantly with the many enabling factors and forces that contributed to maintaining safety at Southmead, demonstrating how intervention and context are co-constructive.

These findings are consistent with, and add to, the body of work on improvement capabilities, defined as the organisational ability to use improvement approaches and methods to enhance performance (Furnival et al., 2017). But our analysis goes beyond the provision of a list of high-level categories linked to safety to offer an empirically grounded description of what these categories look like in practice, how they interact with each other, and what organisational work needs to be done.

The study does have limitations. As the findings are based on a single site, it will be important to explore these mechanisms in other maternity units and acute care settings characterised by different performance, and to develop measurable indicators for profiling maternity units on a larger scale. Our analysis is focused on the “inner context” of a specific clinical microsystem; the “outer contexts” of the wider hospital, policy, and political environment were beyond the scope of our study, but are likely to exert their own important effects – and will be especially relevant in attempts to scale-up in global environments.

## 5 Conclusions

Tempting though it may be, our analysis suggests that foregrounding a specific intervention or programme as the major explanation for safety performance may risk rendering invisible or backgrounding important features of context that are generative of safety. The improvement logics of intervention and context should be understood as a unity, not a duality. Safety, especially in acute care settings such as maternity care, is an emergent property of complex systems: enduring high performance is likely to be dependent on reshaping the multiple, interacting features of systems (including specific interventions) in a way that enables the reproduction of desired outcomes.
